# A key role of PGC‐1*α* transcriptional coactivator in production of VEGF by a novel angiogenic agent COA‐Cl in cultured human fibroblasts

**DOI:** 10.14814/phy2.12742

**Published:** 2016-03-31

**Authors:** Junsuke Igarashi, Ryuji Okamoto, Tetsuo Yamashita, Takeshi Hashimoto, Sakiko Karita, Kozo Nakai, Yasuo Kubota, Maki Takata, Fuminori Yamaguchi, Masaaki Tokuda, Norikazu Sakakibara, Ikuko Tsukamoto, Ryoji Konishi, Katsuya Hirano

**Affiliations:** ^1^ Department of Cardiovascular PhysiologyFaculty of MedicineKagawa UniversityKita‐GunJapan; ^2^ Department of DermatologyFaculty of MedicineKagawa UniversityKita‐GunJapan; ^3^ Department of Pharmaco‐Bio‐InformaticsFaculty of MedicineKagawa UniversityKita‐GunJapan; ^4^ Department of Cell PhysiologyFaculty of MedicineKagawa UniversityKita‐GunJapan; ^5^ Kagawa School of Pharmaceutical SciencesTokushima Bunri UniversitySanukiJapan

**Keywords:** Angiogenesis, gene regulation, nucleic acid, PGC‐1*α*, transcriptional coactivator, VEGF

## Abstract

We previously demonstrated a potent angiogenic effect of a newly developed adenosine‐like agent named COA‐Cl. COA‐Cl exerted tube forming activity in human umbilical vein endothelial cells in the presence of normal human dermal fibroblasts (NHDF). We therefore explored whether and how COA‐Cl modulates gene expression and protein secretion of VEGF, a master regulator of angiogenesis, in NHDF. RT‐PCR and ELISA revealed that COA‐Cl upregulated VEGF mRNA expression and protein secretion in NHDF. HIF1*α* (hypoxia‐inducible factor 1*α*), a transcription factor, and PGC‐1*α* (peroxisome proliferator‐activated receptor‐*γ* coactivator‐1*α*), a transcriptional coactivator, are known to positively regulate the VEGF gene. Immunoblot and RT‐PCR analyses revealed that COA‐Cl markedly upregulated the expression of PGC‐1*α* protein and mRNA. COA‐Cl had no effect on the expression of HIF1*α* protein and mRNA in both hypoxia and normoxia. Silencing PGC‐1*α* gene, but not HIF1*α* gene, by small interfering RNA attenuated the ability of COA‐Cl to promote VEGF secretion. When an N‐terminal fragment of PGC‐1*α* was cotransfected with its partner transcription factor ERR *α* (estrogen‐related receptor‐*α*) in COS‐7 cells, COA‐Cl upregulated the expression of the endogenous VEGF mRNA. However, COA‐Cl had no effect on the expression of VEGF, when HIF1*α* was transfected. COA‐Cl induces VEGF gene expression and protein secretion in fibroblasts. The transcriptional coactivator PGC‐1*α*, in concert with ERR *α*, plays a key role in the COA‐Cl‐induced VEGF production. COA‐Cl‐induced activation of PGC‐1*α*‐ERR *α*‐VEGF pathway has a potential as a novel means for therapeutic angiogenesis.

## Introduction

Ischemic diseases comprise the largest cause of mortality and morbidity worldwide, in association with the westernization of our life style and the progress of industrialization. Angiogenesis is a process in which new blood vessels develop from the preexisting ones. It occurs in response to tissue ischemia, and thereby helps to maintain some degree of blood flow in the ischemic areas. Therapeutic angiogenesis is therefore a potential means to fight against various ischemic disorders. Many challenges have been made for this purpose, including gene therapy, cellular transplantation, and surgical implantation of cellular sheets (Hollander et al. 2014; Ko and Bandyk 2014). These procedures require special techniques and facilities, and have not yet been widely used in clinic.

VEGF is an endogenously present polypeptide growth factor and is a master regulator of angiogenesis (Crafts et al. 2015). In relation to aforementioned therapeutic angiogenesis, Isner and coworkers treated rat hearts exposed to ischemia‐reperfusion with recombinant human VEGF protein, and observed beneficial effects in their cardiovascular functions in 1997 (Luo et al. 1997). Since then treatment with recombinant proangiogenic growth factor proteins including VEGF has yielded promising outcomes in various ischemic situations such as peripheral artery diseases, both in experimental settings and in clinical trials (Ko and Bandyk 2014). However, owing to its expensiveness and molecular instability of the recombinant proteins, it has not been commonly performed in clinical practice at this stage.

We have recently developed a novel nucleic acid analog termed COA‐Cl [6‐amino‐2‐chloro‐9‐[*trans*‐*trans*‐2′,3′‐bis(hydroxymethyl)cyclobuthyl]purine), structurally related to adenosine. COA‐Cl induces strong angiogenic responses in three independent experimental models: in vitro cultured human umbilical vein endothelial cells (HUVEC), as well as in vivo chicken chorioallantoic membrane and rabbit corneal matrigel implant models (Tsukamoto et al. 2010). A G‐protein coupled receptor S1P_1_ appears to mediate some part of the effects of COA‐Cl in HUVEC (Igarashi et al. 2014). However, antagonists of S1P_1_ exhibit incomplete attenuation of HUVEC tube forming activity induced by COA‐Cl, suggesting that COA‐Cl modulates additional proangiogenic mechanism(s) besides activation of endothelial S1P_1_. Furthermore, recent reports demonstrate that endothelial S1P_1_ receptor contributes for proper execution of angiogenesis by seizing endothelial vessel sprouting elicited by VEGF (Gaengel et al. 2012), suggesting that COA‐Cl might induce some sprouting signal before activating endothelial S1P_1_. Interestingly, tube forming activity of COA‐Cl was identified in a coculture model of HUVEC with normal human dermal fibroblasts (NHDF) (Tsukamoto et al. 2010). An inhibitor of VEGF receptor partially attenuated COA‐Cl‐induced endothelial tube forming activity in this coculture model, while COA‐Cl *per se* did not activate the VEGF receptor in HUVEC mono‐culture (Tsukamoto et al. 2010). These results suggest that COA‐Cl induced VEGF expression and secretion in NHDF that in turn may stimulate endothelial cells in a paracrine fashion.

The transcriptional regulation plays a primary role in the regulation of expression and secretion of VEGF. Various transcriptional regulatory factors are involved in both basal and stimulated expression of VEGF (Pages and Pouyssegur 2005). Ischemic insult is one of the representative situations that induce upregulation of VEGF. In this situation, two transcriptional regulators, hypoxia‐inducible factor‐1*α* (HIF1*α*) and peroxisome proliferator‐activated receptor‐*γ* coactivator‐1*α* (PGC‐1*α*), play a major role in upregulating VEGF gene expression (Shoag and Arany 2010). HIF1*α* is a transcription factor and regulates the expression of VEGF in concert with HIF1*β* (Ahluwalia and Tarnawski 2012). PGC‐1*α* is a transcriptional coactivator and regulates the expression of VEGF in concert with a transcription factor estrogen‐related receptor‐*α* (ERR*α*) (Arany et al. 2008).

In this study, we aimed to clarify whether COA‐Cl induces the expression and secretion of VEGF in NHDF, and if so, we also tried to elucidate the underlying mechanisms. This study provides evidence that COA‐Cl promotes VEGF gene expression and protein secretion in NHDF via a transcriptional coactivator PGC‐1*α*.

## Materials and Methods

### Cell culture and drug treatment

NHDF were obtained from Kurabo (Osaka, Japan) (Tsukamoto et al. 2010). They were maintained in culture using *α*‐minimum essential media (Wako, Osaka, Japan) supplemented with 10% FBS (Hyclone, Logan, CT) and penicillin/streptomycin (Life Technologies, Carlsbad, CA) in a humidified incubator at 37°C, perfused with 5% CO_2_. Cells were split at a ratio of 1:6 and were used for experiments between P4 and P9 at day 3 to 4. COS‐7 cells were cultured as previously described (Igarashi et al. 2009). COA‐Cl was synthesized as previously described (Tsukamoto et al. 2010) and was solubilized into 150 mM of NaCl. Stock solutions of other drugs were prepared as instructed by suppliers or as described elsewhere (Tsukamoto et al. 2010; Igarashi et al. 2014). In some protocols, cells were cultured with a hypoxic gas mixture (94% N_2_, 5% CO_2_ and 1% O_2_) using an APM‐30D chamber (ASTEC, Shime, Japan).

### ELISA

NHDF plated on a 12‐well plate were incubated with 500 *μ*L of culture medium that bear various concentrations of COA‐Cl or vehicle. The culture medium was collected and immediately subjected to ELISA by using a commercially available kit specific for human VEGF (R&D systems, Minneapolis, MN) as instructed. The samples were subjected to a duplicate measurement of optical density, and the average of the readings was obtained for each sample. The amount of VEGF secretion was expressed as pg/mL of culture media, according to the readings of the standard samples (15.6 to 1000 pg/mL) provided in the kit.

### RT‐PCR

RNA isolation and conventional RT‐PCR assay were performed as described (Igarashi et al. 2014). Primer sequences of oligonucleotides are summarized in the Table 1. Primer pairs were designed to span at least one intron in order to avoid any amplification of contaminated genome DNA; one exception was primers used to detect *Chlorocebus Sabaeus* VEGF, which were designed within the exon‐1 to detect a common sequence of all six deposited transcript variants.

**Table 1:** Information of RT‐PCR assay

Primer sequence		Amplicon length (bp)	Target exon(s)	GenBank accession
VEGF (*Homo sapiens*)	5′‐CGAAACCATGAACTTTCTGC‐3′	302	1 to 3	NM_003376.5
	5′‐CCTCAGTGGGCACACACTCC‐3′			
GAPDH (*Homo sapiens*)	5′‐ACCACAGTCCATGCCATCAC‐3′	452	7 to 8	NM_002046.5
	5′‐TCCACCACCCTGTTGCTGTA‐3′			
PGC‐1*α* (*Homo sapiens*)	5′‐TTGACTGGCGTCATTCAGGA‐3′	346	1 to 3	NM_013261.3
	5′‐GGGCAATCCGTCTTCATCCA‐3′			
ERR*α* (*Homo sapiens*)	5′‐GGCGGCAGAAGTACAAGC‐3′	116	4 to 5	NM_004451.3
	5′‐ATTCACTGGGGCTGCTGT‐3′			
HIF1*α* (*Homo sapiens*)	5′‐CGCGAACGACAAGAAAAAG‐3′	122	2 to 3	NM_001530.3
	5′‐GAAGTGGCAACTGATGAGCA‐3′			
FGF1 (*Homo sapiens*)	5′‐TGAGAAGAAGACACCAAGTGGA‐3′	110	1 to 2	NM_000800.3
	5′‐TTGTGGCGCTTTCAAGACTA‐3′			
FGF2 (*Homo sapiens*)	5′‐AGCGGCTGTACTGCAAAAAC‐3′	109	1 to 2	NM_002006.4
	5′‐GCTTGAAGTTGTAGCTTGATGTG‐3′			
HB‐EGF (*Homo sapiens*)	5′‐GGCAGATCTGGACCTTTTGA‐3′	107	2 to 3	NM_001945.2
	5′‐CTAGCCCCTTGCCTTTCTTC‐3′			
ANGPT1 (*Homo sapiens*)	5′‐GCTACCATGCTGGAGATAGGA‐3′	109	2 to 3	NM_001146.3
	5′‐TCTCAAGTCGAGAAGTTTGATTTAGT‐3′			
VEGF (*Chlorocebus sabaeus*)	5′‐GACACACCCACCCACATACA‐3′	216	1	XM_007972427‐32
	5′‐TCTCCTCCTCTTCCCTGTCA‐3′			

### Immunoblot (IB) analyses

NHDF lysates were prepared using a buffer system derived from a NE‐PER Nuclear and Cytoplasmic Extraction Reagent kit (Thermo Fisher, Waltham, MA). Cells on a 100‐mm culture dish were washed with PBS, followed by an addition of 250 *μ*L of CERI buffer supplemented with the Protease Cocktail III (Merck, Whitehouse Station, NJ). After being transferred to microfuge tubes, the cell suspensions were vortexed for 15 sec and incubated on ice for 10 min. Following the addition of CERII buffer (13.75 *μ*L), cell lysate was vortexed for 5 sec. Following another period of incubation on ice for 1 min, they were subjected to final vortexing for 5 sec. Resulting protein sample solutions were mixed with an equal amount of 2X Leamlli's protein sample buffer. COS‐7 cell lysates were prepared as described previously (Igarashi et al. 2009).

Equal amounts of cellular samples (typically ~5 *μ*g proteins/lane) were size‐fractionated by SDS‐PAGE, transferred to a nitrocellulose membrane, and was subjected to IB as described elsewhere in detail (Tsukamoto et al. 2010; Igarashi et al. 2014). Primary antibodies used in this study are as follows: a mouse monoclonal antibody specific for mouse PGC‐1*α* amino acids 1‐120 (Trausch‐Azar et al. 2010; Adamovich et al. 2013) (Millipore, San Diego, CA), a rabbit monoclonal antibody specific for human GAPDH (Abcam, Cambridge, MA), a rabbit monoclonal antibody specific for human ERR*α* (Abcam) and a mouse monoclonal antibody specific for human HIF1*α* (BD biosciences, San Jose, CA).

### Transfection with small interfering RNA (siRNA)

NHDF were transiently transfected with 10 nmol/L of siRNA specific for human PGC‐1*α* (Hs_PPARGC1A_5; Qiagen, Valencia, CA) and human HIF1*α* (Hs_HIF1A_7; Qiagen), or AllStars Negative Control siRNA (Sigma, St. Louis, MO), using OptiMEM and Lipofectamine RNAiMAX (Life Technologies) as instructed (Igarashi et al. 2013, 2014).

### Plasmid construction and transient transfection

The cDNA encoding mouse N‐truncated isoform of PGC‐1*α* (NT‐PGC‐1*α*) was obtained by PCR amplification using the cDNA encoding mouse full‐length PGC‐1*α* (FL‐PGC‐1*α*) (Monsalve et al. 2000) as a template (Addgene, Cambridge, MA). A PCR reaction was performed to insert additional intronic residues that are specific for NT‐PGC‐1*α* (Zhang et al. 2009) by using a forward primer: 5′‐GGAGACCCAAGCTGGCTAG‐3′ and a reverse primer: 5′‐GAAGATATTCTAGATTTATAAAAACAAATTTGGTGACTCTGGGGTCA‐3′. The resulting amplicon was double‐digested with *Eco*RV and *Xba*I, and was then subcloned into the pcDNA3.1 (+) vector. A 2480‐bp DNA fragment corresponding to human HIF1*α* (Genbank accession number NM_001530) was PCR‐amplified by using a human cDNA library as a template. The resulting amplicon was double‐digested with *Eco*RV and *Xba*I, and was then subcloned into pME18S‐FLAG vector (Toyobo, Osaka, Japan). The sequences of the protein‐coding regions of the two plasmid constructs were verified to have no unintended mutations. A plasmid construct harboring full‐length human ERR*α* subcloned into pCMFlag vector was purchased from RIKEN BRC DNA Bank (Tsukuba, Japan).

COS‐7 cells on a 60‐mm culture dish were transfected with a total of 4 *μ*g cDNA plasmids along with 10 *μ*L of Lipofectamine 2000 (Lifescience Technologies) as instructed. These experimental protocols had been approved by the Kagawa University Recombinant DNA Experiment Committee.

### Other methods

COA‐Cl was synthesized as described previously (Tsukamoto et al. 2010). Impurities were not detected by nuclear magnetic resonance spectroscopy. Materials not mentioned above were commercially obtained at the highest quality available. All experiments were performed at least three times. Statistical differences were analyzed by analyses of variance (ANOVA) followed by Scheffe's *F*‐test or by Student's *t*‐test where appropriate using Statcel3 software (OMS, Saitama, Japan). All data are expressed as means ± S.E.M. A *P*‐value of <0.05 was considered statistically significant.

## Results

### COA‐Cl induces VEGF mRNA expression and protein secretion in NHDF

**Figure 1:**
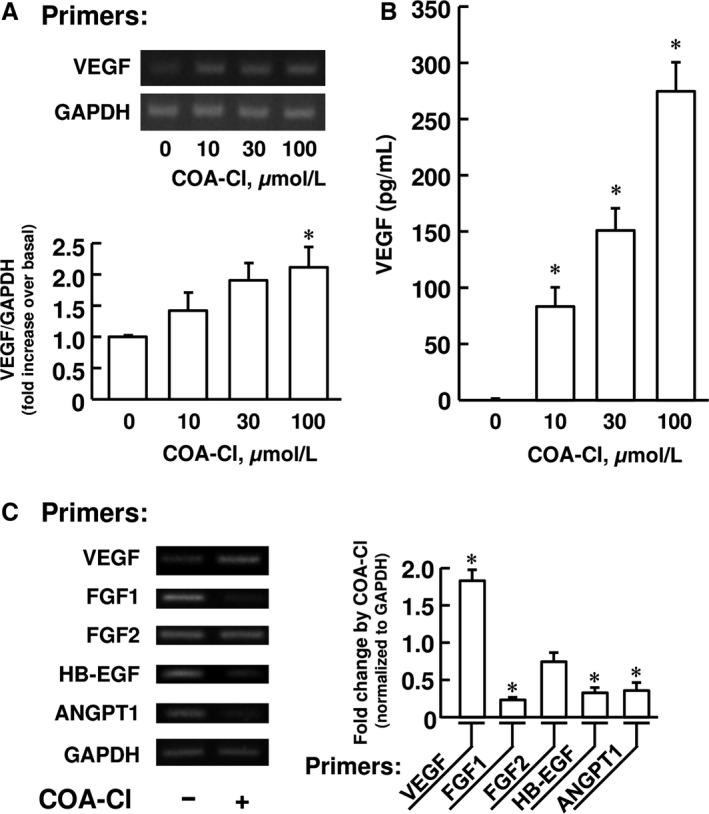
COA‐Cl promotes VEGF mRNA expression and protein secretion into culture medium in NHDF. (A) Representative agarose gel electrophoretogram and summary (*n *= 5) of RT‐PCR analysis of the expression of VEGF and GAPDH mRNA in NHDF treated with the indicated concentrations of COA‐Cl for 48 h. The level of PGC‐1*α* mRNA was normalized to that of GAPDH mRNA, and then expressed as a fold change relative to that obtained in the absence of COA‐Cl. (B) Summary (*n *= 3) of ELISA specific for VEGF using culture medium derived from NHDF treated with indicated concentrations of COA‐Cl. (C) Representative agarose gel electrophoretogram and summary (*n *= 4) of RT‐PCR specific for transcripts encoding pro‐angiogenic growth factors and GAPDH, as indicated, in NHDF treated with 100 μmol/L COA‐Cl for 48 h. Data are expressed as means ± S.E.M. in the graphs A to C. *P < 0.05 versus COA‐Cl (‐) in Panels A to C.

We at first examined whether or not COA‐Cl induces VEGF in NHDF. We treated the cells with increasing concentrations of COA‐Cl for 48 h and then collected cell lysates and culture medium. The former samples were subjected to RNA isolation followed by RT‐PCR assays specific for human VEGF transcript, and the latter to ELISA specific for human VEGF protein. COA‐Cl induced upregulation of VEGF mRNA expression in NHDF and protein secretion of VEGF into the culture medium in a concentration‐dependent manner (Fig. 1A and B). The significant upregulation of mRNA expression and protein secretion were obtained with 100 μmol/L and 10 μmol/L COA‐Cl, respectively. These results are in agreement with earlier observations with regard to the concentrations at which COA‐Cl induces angiogenic responses [10 to 100 μmol/L, (Igarashi et al. 2014; Tsukamoto et al. 2010)]. The fibroblasts are capable of expressing various proangiogenic growth factors besides VEGF (Lieu et al. 2011), and it is hence possible that COA‐Cl induces these molecules as well as VEGF. We indeed detected mRNAs encoding FGF1, FGF2, heparin‐binding EGF (HB‐EGF) and angiopoietin‐1 (ANGPT1) in our RT‐PCR assay using NHDF (Fig. 1C). However, COA‐Cl induced no significant elevation of the expression of FGF1, FGF2, HB‐EGF, or ANGPT1. The expression of FGF1, HB‐EGF, and ANGPT1 was rather significantly downregulated, whereas the expression of FGF2 remained unaffected (Fig. 1C). Thus, among tested, VEGF was the only proangiogenic growth factor gene that was upregulated by COA‐Cl in NHDF.

### Effects of COA‐Cl on the expression of the transcriptional regulatory factors involved in the regulation of the VEGF gene expression in NHDF

**Figure 2:**
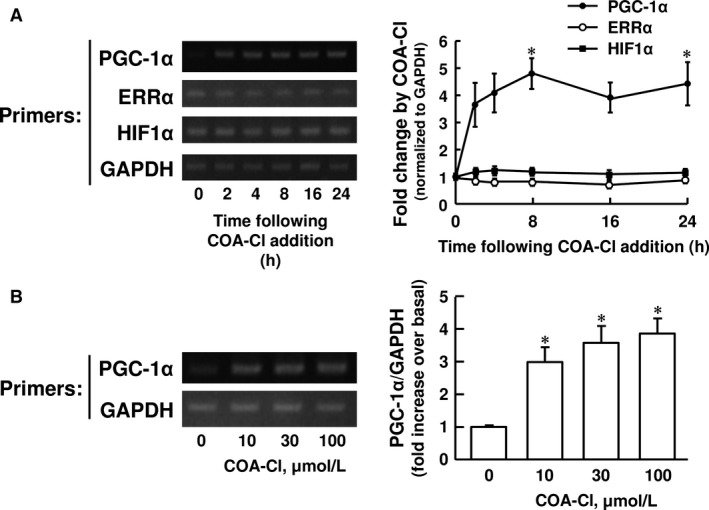
Effects of COA‐Cl on mRNA expression of PGC‐1*α* in NHDF. Representative agarose gel electrophoretograms and summaries of RT‐PCR analysis showing the time‐ (A; *n *= 6) and concentration‐dependent (B; *n *= 5) effects of COA‐Cl on the expression of PGC‐1*α*, ERR *α*, HIF1*α* and GAPDH. NHDF were treated with 100 μmol/L COA‐Cl (A) and for 4 h (B). The level of PGC‐1*α* mRNA was normalized to that of GAPDH mRNA, and then expressed as a fold change relative to that obtained at time 0 (A) and that obtained in the absence of COA‐Cl (B). Data are expressed as means ± S.E.M. in the graphs A and B. *P < 0.05 versus time 0 (A) and COA‐Cl (‐) (B).

The activation of transcription plays an important role in regulating the biological activity of VEGF (Pages and Pouyssegur 2005). PGC‐1*α* and HIF1*α* represent the major transcriptional pathways that regulate the VEGF gene expression (Arany et al. 2008; Ahluwalia and Tarnawski 2012). We therefore sought to explore whether or not COA‐Cl modulates these two transcriptional pathways. We examined the effects of COA‐Cl on the expression levels of PGC‐1*α* and HIF1*α* with RT‐PCR analysis (Fig. 2). COA‐Cl (100 *μ*M) elevated the expression of PGC‐1*α* mRNA at 8 h, and the increased level persisted as long as 24 h after COA‐Cl stimulation (Fig. 2A). The significant upregulation of PGC‐1*α* mRNA was observed at 10 *μ*M and higher concentrations of COA‐Cl (Fig. 2B). On the other hand, HIF1*α* mRNA expression levels stayed unchanged over 24‐h treatment with 100 μmol/L COA‐Cl (Fig. 2A). ERR*α* is a nuclear receptor and a transcription factor, which regulates the enhancer elements of VEGF gene in concert with PGC‐1*α* (Arany et al. 2008). The level of ERR*α* mRNA expression, however, remained unchanged after COA‐Cl treatment (Fig. 2A).

**Figure 3:**
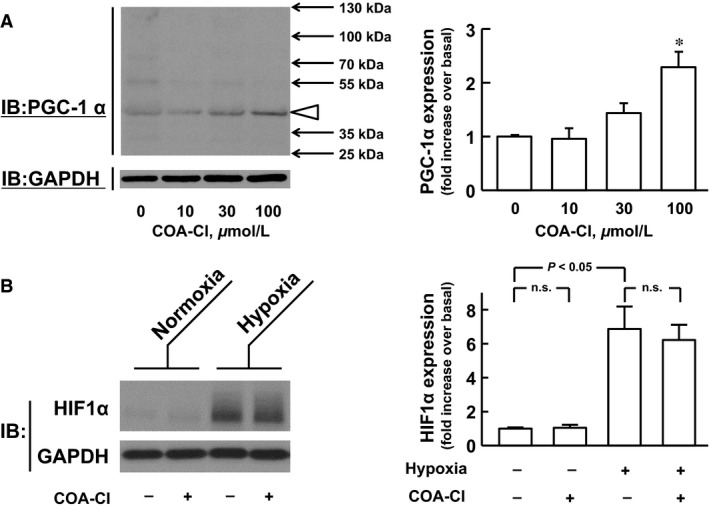
COA‐Cl elevates protein expression of PGC‐1*α*, but not HIF1*α*, in NHDF. (A) Representative immunoblot (IB) images and summary (*n *= 4) for the concentration‐dependent effect of COA‐Cl on the PGC‐1*α* protein expression. NHDF were treated for 24 h with the indicated concentrations of COA‐Cl under normoxic condition. (B) Representative immunoblot images and summary (*n *= 4) showing the effect of 24‐h treatment with 100 *μ*mol/L COA‐Cl on the expression of HIF1*α* under normoxic and hypoxic conditions. NHDF were exposed to hypoxic condition during the last 3 h of COA‐Cl treatment period. The levels of PGC‐1*α* (A) and HIF1*α* (B) were normalized to those of GAPDH, and then expressed as a fold increase relative to those obtained without COA‐Cl treatment under normoxic condition. Data are expressed as means ± S.E.M. in the graphs A and B. *P < 0.05 versus COA‐Cl (‐) (A). n.s. not significant (B).

There are at least seven isoforms of PGC‐1*α* protein in mammalian cells, which arise by multiple alternative splicing as well as alternative promoter (Chan and Arany 2014). In general, PGC‐1*α* protein expression level is low in various cell types, while the protein is extremely unstable because of both high activity in its degradation machineries (Sano et al. 2007) and its own “intrinsically disordered” properties (Adamovich et al. 2013). The antibody used in this study is raised against the N‐terminal region of PGC‐1*α* and therefore detects both full‐length (FL‐) and N‐truncated (NT‐) isoforms (Zhang et al. 2009). The immunoblot analysis detected a band with an apparent molecular mass of ~40 kDa in NHDF, but not a band with a molecular mass of ~120 kDa (Fig. 3A). The 40‐kDa band is suggested to represent the NT‐PGC‐1*α* isoform, according to the earlier observations (Zhang et al. 2009; Thom et al. 2014). The level of NT‐PGC‐1*α* increased in a concentration‐dependent fashion 24 h after COA‐Cl treatment, while a significant increase was observed with 100 μmol/L (Fig. 3A). In contrast, the expression of HIF1*α* protein substantially increased after the exposure of NHDF to hypoxic condition. However, COA‐Cl had no effect on the expression levels of HIF1*α* protein in either normoxic or hypoxic conditions (Fig. 3B).

### Effects of gene silencing and overexpression of PGC‐1*α* and HIF1*α* on the induction of VEGF by COA‐Cl

**Figure 4:**
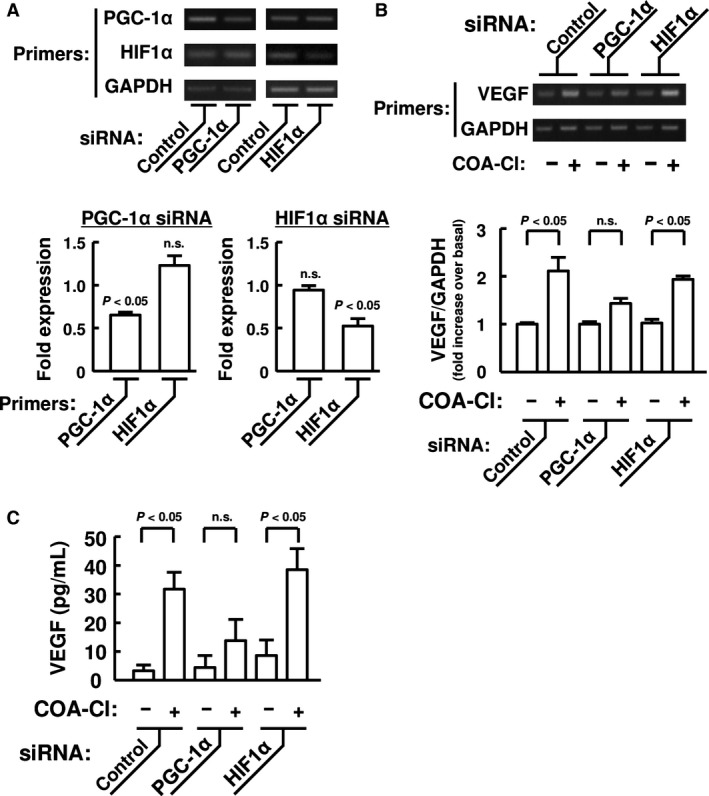
Effects of PGC‐1*α* and HIF1*α* gene silencing on VEGF expression and secretion in NHDF. (A) Representative agarose gel electrophoretograms and summary (*n *= 5) showing the efficacy and specificity of siRNA‐mediated silencing of PGC‐1*α* and HIF1*α* mRNA expression. NHDF were transfected with 10 nmol/L siRNA, and the levels of PGC‐1*α*, HIF1*α* and GAPDH mRNA were evaluated 48 h after transfection. The levels of PGC‐1*α* and HIF1*α* were normalized to those of GAPDH, and then the levels seen in the cells transfected with the gene‐targeted siRNA were expressed as a fold change relative to those seen with control siRNA. n.s. not significant versus control siRNA. (B, C) Representative agarose electrophoretograms and summaries showing the effects of gene silencing of PGC‐1*α* and HIF1*α* on the expression of VEGF mRNA (B; *n *= 4) and the secretion of VEGF protein in the culture media (C; *n *= 6). Thirty‐two hours after transfection with the indicated siRNA, NHDF were treated with 100 μmol/L of COA‐Cl for 16 h (B) and 48 h (C). The levels of VEGF mRNA were normalized to those of GAPDH, and then expressed as a fold change relative to those obtained with control siRNA and without COA‐Cl treatment. Data are expressed as means ± S.E.M. in the graphs A to C. n.s. not significant versus COA‐Cl (‐).

In order to consolidate the functional role of PGC‐1*α*, the effects of the gene silencing of PGC‐1*α* and HIF1*α* on COA‐Cl‐promoted VEGF expression and secretion were investigated (Fig. 4). Transfection of siRNA caused a specific knock‐down of the mRNA expression of the target genes by approximately 50% (Fig. 4A). When the expression of PGC‐1*α* was suppressed, the significant increase in the VEGF mRNA expression and protein secretion by COA‐Cl were eliminated (Fig. 4B and 4C). However, the suppression of HIF1*α* had no effect on the increase in the VEGF mRNA expression and protein secretion by COA‐Cl (Fig. 4B and 4C). However, the degrees of decreases in VEGF mRNA expression (Fig. 4B) and protein secretion (Fig. 4C) in COA‐Cl‐treated cells did not reach the statistical significance, respectively.

**Figure 5:**
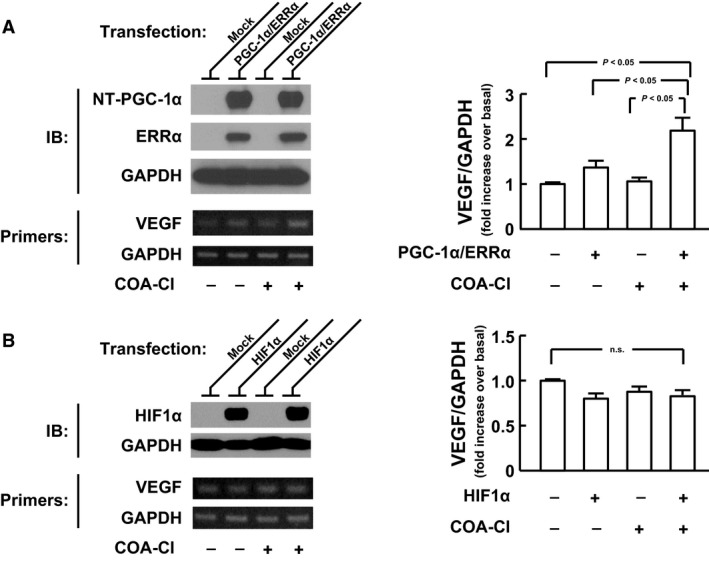
Effects of COA‐Cl on VEGF mRNA expression in COS‐7 cells expressing exogenous NT‐PGC‐1*α*, ERR *α* or HIF1*α*. Representative agarose gel electrophoretograms and summaries showing the effect of COA‐Cl on the expression of VEGF mRNA expression in COS‐7 cells expressing NT‐PGC‐1*α* and ERR *α* (A; *n *= 4) and HIF1*α* (B; *n *= 3). Representative immunoblots (IB) images verify the comparable levels of expression of exogenous NT‐PGC‐1*α*, ERR *α* or HIF1*α* with and without COA‐Cl treatment. Thirty‐two hours after transfection, cells were treated with 100 μmol/L COA‐Cl or vehicle for 16 h. The levels of VEGF were normalized to those of GAPDH, and then expressed as a fold change relative to those obtained with mock transfection and without COA‐Cl treatment. Data are expressed as means ± S.E.M. in the graphs A and B. n.s., not significant.

The functional relevance of NT‐PGC‐1*α*‐dependent mechanism in the COA‐Cl‐induced induction of VEGF gene was investigated in a heterologous expression system. For this sake, we utilized COS‐7 fibroblast like cells that exert high plasmid DNA transfection efficiency (Igarashi et al. 2009). COS‐7 cells were transiently cotransfected with mouse NT‐PGC‐1*α* and human ERR*α*. In the control COS‐7 cells without any expression of the exogenous NT‐PGC‐1*α* and ERR*α*, COA‐Cl failed to increase the expression of VEGF mRNA (Fig. 5A). The over‐expression of NT‐PGC‐1*α* and ERR*α*,* per se*, had no effect on the basal expression of VEGF mRNA (Fig. 5A). When stimulated with 100 *μ*M COA‐Cl, the expression of VEGF mRNA was significantly upregulated in the cells coexpressing NT‐PGC‐1*α* and ERR*α* (Fig. 5A). In contrast, COA‐Cl had no effect on the expression of VEGF mRNA in cells over‐expressing human HIF1*α* (Fig. 5B).

## Discussion

This study demonstrates that COA‐Cl, a recently developed proangiogenic adenosine analog, elevates mRNA expression and protein secretion of VEGF, the best‐characterized proangiogenic growth factor, in NHDF. The induction of the expression by COA‐Cl appears to be specific to VEGF among other possible proangiogenic growth factors, including FGF1, FGF2, HB‐EGF, and ANGPT1. The induction of the VEGF secretion is therefore suggested to contribute to the angiogenic activity of COA‐Cl, especially that previously observed in the coculture system of endothelial cells and fibroblasts (Tsukamoto et al. 2010; Igarashi et al. 2014). Furthermore, this study demonstrated that the induction of VEGF by COA‐Cl is dependent on a transcriptional coactivator PGC‐1*α*. COA‐Cl upregulated the expression of PGC‐1*α*, while gene silencing of PGC‐1*α* abrogated induction of VEGF expression and secretion by COA‐Cl. Thus, this study identifies another mechanism by which COA‐Cl may promote angiogenic responses, that is, PGC‐1*α*‐mediated induction of VEGF in the fibroblast that in turn may stimulate the vascular endothelial cells.

During the process of angiogensis, vascular endothelial cells initially sprout in response to growth factors like VEGF, and then newly formed endothelial tubes gradually maturate in response to various bioactive molecules including a lipid mediator sphingosine 1‐phosphate (S1P) (Jain 2003). In seeking to explore the mechanisms how COA‐Cl might promote angiogenic responses, we have previously utilized HUVEC mono‐culture. We have found that COA‐Cl activates MAP kinases ERK1/2 in HUVEC, apparently involving the G‐protein coupled S1P_1_ receptor (Igarashi et al. 2014). However, the S1P/S1P_1_ pathway does not initiate angiogenic responses by itself, but rather induces maturation of newly formed endothelial tubes (Gaengel et al. 2012). This study now indicates that COA‐Cl markedly induces VEGF in NHDF monoculture. We therefore propose that COA‐Cl may exert two distinct mechanisms of action to facilitate angiogenic responses: promotion of VEGF secretion from fibroblasts that stimulates endothelial VEGF receptor leading to endothelial sprouting, and direct stimulation of S1P_1_ receptors in the vascular endothelial cells associated with maturation of endothelial sprouts.

PGC‐1*α* was originally identified as a transcriptional coactivator that links cold exposure to adaptive thermogenesis response in brown fat and skeletal muscle (Puigserver et al. 1998). PGC‐1*α* has emerged as a novel regulator of the VEGF gene (Arany et al. 2008). PGC‐1*α* regulates a wide array of transcription factors to induce its biological effects such as mitochondrial biogenesis (Ventura‐Clapier et al. 2008). Among the transcription factors that are activated by PGC‐1*α*, ERR*α* is specifically engaged in the regulation of the transcription of the VEGF gene, thereby contributing to VEGF‐evoked angiogenesis (Arany et al. 2008; Thom et al. 2014). On the other hand, a transcription factor HIF1*α* is well‐established as a regulator of the VEGF gene (Ahluwalia and Tarnawski 2012). HIF1*α* plays a key role in the induction of VEGF gene expression under hypoxic conditions (Pugh and Ratcliffe 2003). Hypoxia elevates the expression of HIF1*α* mainly by preventing protein degradation (Semenza 2001). Nevertheless, some level of HIF1*α* is expressed even under normoxic conditions in some types of cells (Ader et al. 2009; Michaud et al. 2009). In this study, NHDF indeed expressed detectable levels of PGC‐1*α*, ERR*α* and HIF1*α*, with respect to either mRNA or protein expression. However, COA‐Cl specifically upregulated the expression of PGC‐1*α*. COA‐Cl had no effect on the HIF1*α* expression under both normoxic and hypoxic conditions. Gene silencing of PGC‐1*α*, but not HIF1*α*, attenuated the ability of COA‐Cl to elevate VEGF mRNA expression and protein secretion in NHDF. Conversely, COA‐Cl upregulated endogenous VEGF mRNA expression in COS‐7 cells when they overexpress NT‐PGC‐1*α* along with its partner ERR*α*; however, it had no effect when the cells overexpress HIF1*α*. Together, these results point out the PGC‐1*α* in concert with the ERR*α*, rather than the HIF1*α*, as a major pathway that mediates the COA‐Cl‐induced increase in the expression and secretion of VEGF in NHDF. At this stage, it remains to be elucidated whether or not and how COA‐Cl may modulate the PGC‐1*α*/ERR*α*/VEGF axis in a hypoxic condition. Although COA‐Cl promotes angiogenesis in two independent in vivo models, chicken chorioallantoic membrane and rabbit cornea (Tsukamoto et al. 2010), it remains to be explored whether or not COA‐Cl induces VEGF in living animals.

It is of note that a band with an apparent molecular mass of ~40 kDa, which may correspond to the NT‐PGC‐1*α* protein isoform, was only detected as an isoform of PGC‐1*α*, by the immunoblot assays using a previously characterized monoclonal antibody specific for N‐terminal peptide sequence of human PGC‐1*α* (Zhang et al. 2009; Adamovich et al. 2013). The molecular mass of FL‐PGC‐1*α* protein is estimated to be ~90 kDa according to the amino acid sequences, whereas the apparent molecular mass according to the mobility on SDS‐PAGE is estimated to be ~120 kDa (Zhang et al. 2009; Adamovich et al. 2013). However, any signals corresponding to the FL‐PGC‐1*α* were not detected in NHDF. The rat brain and heart also preferentially express the NT‐PGC‐1*α* isoform (Zhang et al. 2009). A recent study identified hypoxic insult switches expression pattern of PGC‐1*α* to a more NT‐type‐oriented one in skeletal muscle, leading to promotion of VEGF expression and secretion (Thom et al. 2014). The major isoforms of PGC‐1*α* are suggested to differ depending on the species and type of tissues, or experimental conditions. However, it remains to be clarified how the NT‐PGC‐1*α* isoforms is specifically expressed in NHDF and upregulated by COA‐Cl.

To our knowledge, our data now raise the first example of a small molecule agent that increases the expression levels of PGC‐1*α*, thereby inducing the expression and secretion of VEGF. On this regard, it is interesting to note that baicalin, a component of traditional Chinese medicine, upregulates the expression level of ERR*α*, but not PGC‐1*α*, in U251 human glioma cell line, thereby inducing the expression of the VEGF gene (Zhang et al. 2011). In this study, COA‐Cl upregulated the expression of PGC‐1*α*, but not ERR*α* mRNA, and thereby induced the expression levels of VEGF in NHDF. It is therefore suggested that the increase in the expression level of either PGC‐1*α* or ERR*α*, whereas the other level remains unchanged, is sufficient for upregulating the expression of VEGF. Regulatory mechanisms for the PGC‐1*α* gene have been principally explored in skeletal muscle and in fat tissue. Several transcription factors were identified as positive regulators of the PGC‐1*α* gene, including myocyte enhancer factor 2 (MEF2), forkhead box class‐O (FoxO1), activating transcription factor 2 (ATF2), and cAMP response element‐binding protein (CREB). These factors are in turn modulated by various upstream signaling modules, involving the calcium/calmodulin and the cAMP pathways (Fernandez‐Marcos and Auwerx 2011). The mechanisms how COA‐Cl elevates PGC‐1*α* gene expression in NHDF remain to be investigated.

In conclusion, we herein demonstrate that COA‐Cl, a novel proangiogenic adenosine‐like compound, promotes expression and secretion of VEGF in cultured human fibroblasts. Significantly, it elevates the expression of a transcriptional coactivator PGC‐1*α*, which in turn activates the transcription of the VEGF gene. Thus, COA‐Cl not only stimulates endothelial S1P_1_ receptors, but also promotes secretion of VEGF from adjacent fibroblasts. We anticipate that a better understanding of the actions of COA‐Cl in both endothelial‐ and in nonendothelial cells would lead to an identification of additional point of control for angiogenesis.

## Conflict of Interest

Nothing.
